# Mapping the DNA-Binding Motif of Scabin Toxin, a Guanine Modifying Enzyme from *Streptomyces scabies*

**DOI:** 10.3390/toxins13010055

**Published:** 2021-01-13

**Authors:** Maritza Vatta, Bronwyn Lyons, Kayla A. Heney, Taylor Lidster, A. Rod Merrill

**Affiliations:** 1 Department of Molecular and Cellular Biology, University of Guelph, Guelph, ON N1G 2W1, Canada; mvatta@uoguelph.ca; 2 Department of Biochemistry and Molecular Biology and Center for Blood Research, University of British Columbia, 2350 Health Sciences Mall, Vancouver, BC V6T 1Z3, Canada; lyonsb819@gmail.com; 3 Department of Biochemistry, McGill University, Montreal, QC H3G 1Y6, Canada; kayla.heney@mail.mcgill.ca; 4 Department of Biological Sciences, Brock University, St. Catherines, ON L2S 3A1, Canada; taylorlidster94@gmail.com

**Keywords:** bacterial toxins, crystallography, enzyme kinetics, DNA-binding motif, bioinformatics, DNA modification, molecular modeling

## Abstract

Scabin is a mono-ADP-ribosyltransferase toxin/enzyme and possible virulence factor produced by the agriculture pathogen, *Streptomyces scabies*. Recently, molecular dynamic approaches and MD simulations revealed its interaction with both NAD^+^ and DNA substrates. An Essential Dynamics Analysis identified a crab-claw-like mechanism, including coupled changes in the exposed motifs, and the R_β1_-R_La_-N_Lc_-STT_β2_-W_PN_-W_ARTT_-(QxE)_ARTT_ sequence motif was proposed as a catalytic signature of the Pierisin family of DNA-acting toxins. A new fluorescence assay was devised to measure the kinetics for both RNA and DNA substrates. Several protein variants were prepared to probe the Scabin-NAD-DNA molecular model and to reveal the reaction mechanism for the transfer of ADP-ribose to the guanine base in the DNA substrate. The results revealed that there are several lysine and arginine residues in Scabin that are important for binding the DNA substrate; also, key residues such as Asn110 in the mechanism of ADP-ribose transfer to the guanine base were identified. The DNA-binding residues are shared with ScARP from *Streptomyces coelicolor* but are not conserved with Pierisin-1, suggesting that the modification of guanine bases by ADP-ribosyltransferases is divergent even in the Pierisin family.

## 1. Introduction

Mono-ADP-ribosyltransferase (mART) toxins are a class of enzymes produced by pathogenic bacteria as virulence factors, and are potential targets for developing anti-virulence compounds against their activity [1]. Pathogenic bacteria that utilize mART toxins to inflict toxicity can infect humans (*Vibrio cholerae*, *Bordetella pertussis*), insects (*Bacillus sphaericus*), and even plants (*Pseudomonas syringae*) [2,3,4]. mART toxins catalyze the scission of the glycosidic linkage between the nicotinamide and ADP-ribose of NAD^+^. The bond cleavage is followed with the transfer of the ADP-ribose moiety to a target molecule within a host cell [1,5]. Glycohydrolase (GH) activity is characteristic of most mART toxins, whereby in the absence of a transferase substrate, an OH^−^ molecule in solution acts as a nucleophile to accept the ADP-ribose moiety from NAD^+^ [6]. The adduct from the transferase reaction causes a change in function of the target molecule to either activate, repress, or completely inactivate the target. The target molecule is most often an essential protein for eukaryotic cellular function, such as eukaryotic elongation factor 2 (eEF2), Rho, and actin [7,8,9,10]. Post-translational modification of these proteins often causes host cell apoptosis, as the cell cannot survive without the proper function of these proteins. Within the target protein, the mART toxin labels a specific nucleophilic residue, such as diphthamide (a post-translationally modified His residue), Arg, Asn, Cys, Thr, or Gln [11,12,13,14,15].

The development of the common scab disease in root and tuber vegetables is caused by the soil-dwelling, filamentous bacterium, *Streptomyces scabies* [16]. The disease is characterized by deep-pitted and corky lesions that can cover the entire surface of the vegetable, making it unattractive to consumers [17]. This leads to significant food waste and serious financial loss within the agricultural industry [18]. The development of common scab disease within the host plant is poorly understood and no viable treatment strategy has yet been developed. Once a field is contaminated, it can be very costly to eradicate the *Streptomyces* organism since it forms highly stable spores that infiltrate the soil [19]. *S. scabies* produces a 200-residue, 22-kDa protein that is a single-domain enzyme called Scabin [20,21]. Scabin has been cloned, expressed, and purified, and shown to harbor dual enzymatic activities (GH and ADP-ribosyltransferase). Scabin targets DNA as a substrate for ADP-ribose modification; it also shows enzyme specificity towards genomic DNA from *Solanum tuberosum* tubers [20]. Scabin shares nearly 40% sequence identity with the Pierisin family of mARTs [20]. The Pierisin family members function to couple ADP-ribose to the guanine base in nucleotides and DNA, causing host apoptosis [2,22]. A toxin–antitoxin system known as DarT has been shown to modify the thymine bases in single-stranded DNA [23], and has recently been shown to catalyze reversible ADP-ribosylation [24]. A DNA-targeting enzyme, CARP-1, also was identified from several edible clams and shows similar activity to Pierisins [25,26]. *Streptomyces coelicolor* also produces two DNA-targeting ADP-ribosyltransferases, SCO5461 [27] and ScARP [28]. Scabin represents a mART of bacterial origin that modifies DNA as its target substrate [20], but its role in *S. scabies* virulence is currently unknown.

Scabin was shown to be a more active enzyme (GH activity) compared to most mART toxins. Notably, Scabin exhibited sigmoidal kinetics with substrates such as deoxyguanosine, which differs from the usual Michaelis-Menten behavior of mART toxins. Previously, we determined the crystal structure of apo-Scabin in complex with two competitive inhibitors of the NAD^+^ substrate. A Scabin-NAD^+^-DNA model was built to guide future experiments, including mutagenesis of the active-site scaffold [20].

Furthermore, we investigated the catalytic mechanism of Scabin and characterized the binding of DNA to the enzyme [29]. We determined the Scabin-NADH crystal structure and showed that NADH is a potent competitive inhibitor against the NAD^+^ substrate for this enzyme. This complex provided new insights into the reaction mechanism and facilitated the interpretation of the kinetic variants of the enzyme. Furthermore, we determined the kinetic aspects of model DNA substrates and their modification by Scabin. Importantly, Scabin prefers double-stranded DNA, containing a single base overhang. These results provided a mechanism for DNA-binding to the Scabin enzyme and produced a new DNA-binding motif for this mART toxin [29].

Several crystal structures of Scabin in the presence and absence of competitive NAD^+^ inhibitors and Trp variants were previously determined [20,29]. The Scabin structure has a mART-fold and harbors the conserved R-S-Q-X-E motif. Scabin has an approximately 32% α/β structure and, unlike most mART enzymes, has a large amount of well-ordered coiled structures [20]. Furthermore, Scabin shares low sequence similarity with most mART family members, except for the Pierisin family, and radically differs from well-known mART toxins such as *iota* and the C3-group members, which exhibit high helicity near the N-terminus [30]. Scabin has strong structural homology with the mosquitocidal toxin (MTX) from *Bacillus sphaericus* [31], Pierisin-1, an apoptotic toxin from the cabbage butterfly, *Pieris rapae* [32], and the recently determined structure of ScARP [28]. In general, the involvement of the ARTT- and PN-loops in substrate recognition and catalysis are appreciated for mART toxins [33,34]. However, the newly identified α_1_-α_2_ motif has not been considered for its role in the reaction mechanism for the mART toxins [20,32].

In a recent study, we employed hydrogen-deuterium exchange (HDX), theoretical approaches, molecular dynamics (MD) simulation, and Gaussian Network Modeling (GNM) [35,36] to reveal the dynamic aspects of the Scabin protein in the Scabin-NAD^+^-DNA model [37]. HDX experiments for the Scabin toxin with and without substrates revealed the dynamic aspects of the enzyme and its DNA substrate-binding “footprint”. MD simulations compared the Scabin solution conformation with the static crystal structure. Essential Dynamics Analysis (EDA) and GNM provided a dynamic profile of the enzyme with its crab-claw-like function in its two topological domains. The “crab-claw” dynamics resembles the molecular movements of the C3-like toxins and is a property of the β-scaffold found in the center of catalytic single-domain toxins. The surface exposure and mobile nature of the *cis* side motifs in the Scabin β-core reveal involvement in DNA substrate binding. A novel, Scabin-NAD^+^-DNA ternary model was developed using a docking approach. The sequence motif R_β1_-R_La_-N_Lc_-STT_β2_-W_PN_-W_ARTT_-(QxE)_ARTT_ was touted as a catalytic signature for DNA-acting toxins [37].

The structure of the founding member of the Pierisin family was previously determined with and without the NAD^+^ substrate and of the autoinhibitory form [32]. Pierisin-1 has an N-terminal catalytic and C-terminal ricin B-like domains and, upon modification of the DNA duplex, causes apoptosis in various cancer cells. The GDP substrate recognition and ADP-ribosylation mechanism in the Pierisin family was recently reported for ScARP from *Streptomyces coelicolor* [28]. The structures of ScARP bound to NADH and its GDP substrate were determined at 1.50 and 1.57 Å, respectively. The structure of ScARP with NADH and GDP showed that the guanine base is sandwiched between Trp-59 and the N-ribose of NADH. Notably, H-bonds formed from the N^2^ and N^3^ of the guanine with the Gln-162 OE1 and NE2 atoms, respectively; also, the ADP-ribosylating toxin-turn-turn loop (ARTT-loop), which includes Trp-159 and Gln-162, provides substrate-recognition specificity.

In the present work, we developed a fluorescence-based kinetic assay to measure the ADP-ribose transfer mediated by the Scabin toxin/enzyme to the guanine base of various nucleotides, including ss- and ds-DNA and ss-RNA. This assay has general utility for the study of other DNA-specific ADP-ribosyltransferases. To test our previous Scabin-NAD-DNA model, we prepare several catalytic variants of the active-site core, including key DNA-substrate binding and catalytic residues. The sites where residue substitutions were introduced into the Scabin protein for this study include the α_2_ helix, the L_c_-loop, β_2_, the PN-loop, the ARTT-loop, and the β_6/7_ turn. These results provide the basis for a revised model of the Scabin-NAD-DNA complex based on the kinetic findings for these active-site variants, which leads to an enhanced understanding of the enzyme reaction mechanism shared by the Pierisin family members.

## 2. Results and Discussion

### 2.1. Scabin Sequence-Structure Features

**Figure 1:**
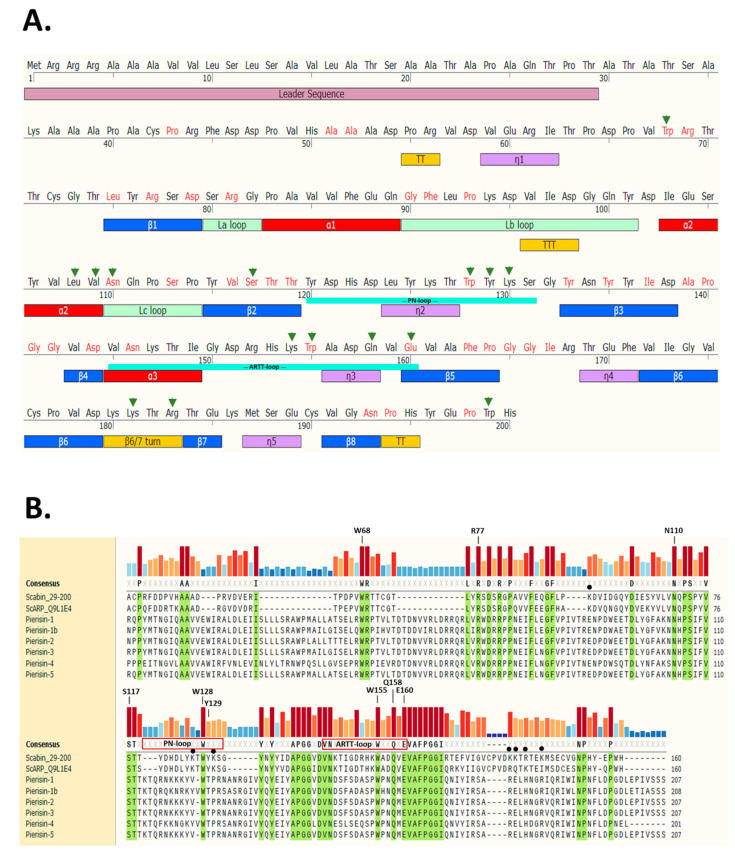
Scabin sequence profile. (**A**) Full-length Scabin sequence showing the leader sequence (salmon), TT/TTT (turns, yellow), n1 (3_10_ helix, mauve), βi (beta strands, blue), αi (helices, red), and Lx (loops, olive green). (**B**) Multiple sequence alignment of Scabin with the Pierisin-family toxins was produced using Muscle [38] and generated using SnapGene ver 5.2 (San Diego, CA). Identical residues shared among the Pierisin family members are highlighted in green and a conservation histogram is shown above the sequence according to color and bar height. The important residues for DNA binding in Scabin are shown with a filled black circle. The PN- and ARTT-loop sequences are shown for the Scabin sequence and the important catalytic sites are indicated (except for W68, which is a structural residue). The Uniprot (https://www.uniprot.org/) identifiers for the sequences are as follows: Scabin (C9Z6T8), ScARP (Q9L1E4), Pierisin-1 (H3JU00), Pierisin-1b (E7EKM3), Pierisin-2 (Q9GV36), Pierisin-3 (C6L2F5), Pierisin-4 (C6L2F6), and Pierisin-5 (A0A0H3V1I0).

Scabin toxin consists of a single catalytic mART (ADP-ribosyltransferase fold) domain and a 29-residue N-terminal secretion signal (Figure 1A).

The secondary structure elements are based on the apo-Scabin crystal structure (PDB: 5DAZ) and the two important catalytic signature loops, PN-loop (Y120-G132) and ARTT-loop (V145-E160), are shown below the sequence. The sites where residue substitutions were introduced into the Scabin protein for this study include the α_2_ helix, the L_c_-loop, β_2_, the PN-loop, the ARTT-loop, and the β_6/7_ turn. The substitution of Trp199 served as a control and is located near the C-terminus outside of the ordered secondary structure. Trp68 is also outside the catalytic core; this structurally important and conserved Trp residue is in a large region between a small 3_10_ helix (n1) and β1 (Figure 1A). A multiple sequence alignment using the Muscle method [38] is depicted in Figure 1B with the identically conserved residues within the Pierisin family highlighted in green. The catalytic signature is highly conserved among all known members of the Pierisin family. The important residues for catalytic activity are labeled above the conservation histogram, except for Trp68, which is an important residue for folding but not catalytic activity. Scabin has some common catalytic features with other members of the mART toxin family. This includes the key Arg (Arg77), which is important for NAD^+^ binding to the active site; the Ser-Thr-Thr motif (Ser117-Thr118-Thr119), which comprises the scaffold of the NAD^+^ binding site [21]; and the hallmark catalytic Gln-X-Glu motif (Gln158-X-Glu160) (Figure 1B). The multiple-sequence alignment shows that Scabin is highly related to the Pierisin mART toxin subgroup, as the catalytic core with its key active-site residues is shared by Scabin and the Pierisin members. A percent identity matrix of several members of the mART toxin family, including Scabin, showed that Scabin shares 40% sequence identity with the Pierisin family members [29] and shares 74% identity with ScARP [28]. The Pierisin group of mART toxins is notable by its target specificity, featuring ADP-ribose transfer to the guanine base in small guanine-containing nucleosides/nucleotides and/or DNA causing apoptosis (Pierisin-1 from *Pieris rapae*) [4] (Figure 1B).

### 2.2. Scabin Structure with Sequence Motifs

**Figure 2:**
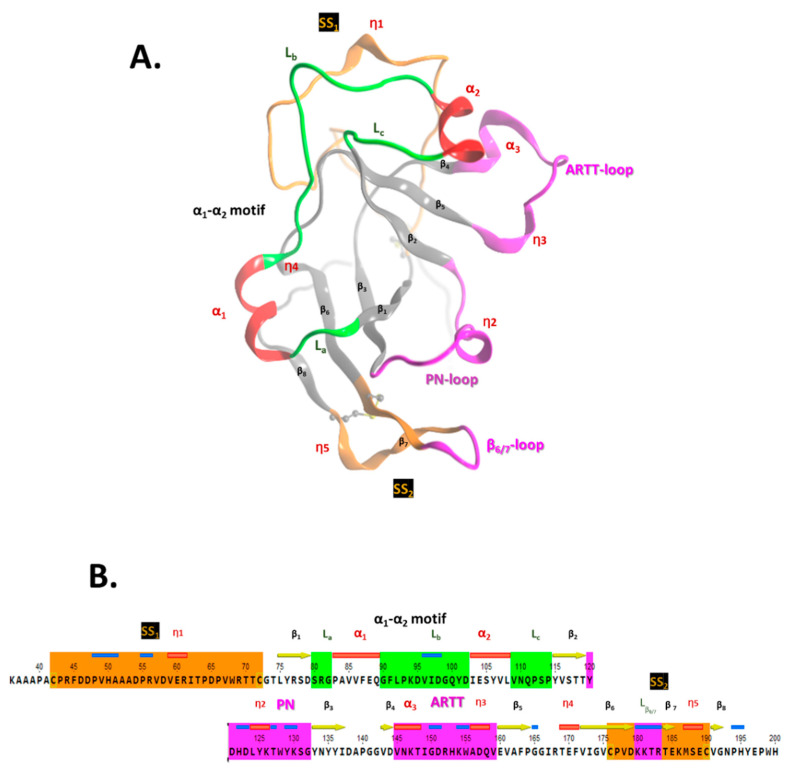
Scabin 3D-topology and sequence. (**A**) Ribbon drawing of Scabin X-ray structure (PDB: 5DAZ) with the following nomenclature: α_i_ for alpha-helix, η_i_ for 3_10-_helix, and β_i_ for β-strand secondary-structure elements. The PN-loop (Y120-G132), ARTT-loop (V145-E160), and β_6/7_-turn (K180-R184) are shown in fuchsia; the SS_1_-loop (C42-C72) and SS_2_-loop (C176-C190) in amber; and the α_1_-α_2_ motif (S80-P114) is rendered with the α-helices in red and coiled segments in green. (**B**) The Scabin catalytic core sequence is shown with corresponding color matches to the X-ray ribbon representation shown in (**A**).

The crystal structure of apo-Scabin was previously solved to a 1.5 Å resolution by Lyons et al. (2016) [20] (PDB: 5DAZ). Scabin consists of a mixed α/β-fold, with a β-sheet core formed by a four-stranded β_I_-sheet (β_1_, β_3_, β_6_, and β_7_-β_8_) perpendicular to a three-stranded β_II_-sheet (β_2_, β_4_, and β_5_), typically observed for the β-fold in mART toxin catalytic domains (Figure 2A). The ARTT-loop (ADP-ribosyl-turn-turn loop) is adjacent to the NAD^+^-binding pocket (*cis* side) and connects β_4_ with β_5_ within the β_I_-sheet, and the β_6/7_-turn links β_6_ with β_7_-β_8_ in the β_II_-sheet. Additionally, two *cis* segments connect the two β-sheets; these are the PN-loop (phosphate-nicotinamide loop) that connects β_2_ with β_3_, and the α_1_-α_2_ motif that tie β_1_ with β_2_. The α_1_-α_2_ motif comprises coiled (L) and helical (α/3_10_) structural elements that alternate with the L_a_-α_1_-L_b_-α_2_-L_c_ pattern in the Scabin primary sequence (Figure 2B).

### 2.3. Scabin-NAD^+^ Interactions

**Figure 3:**
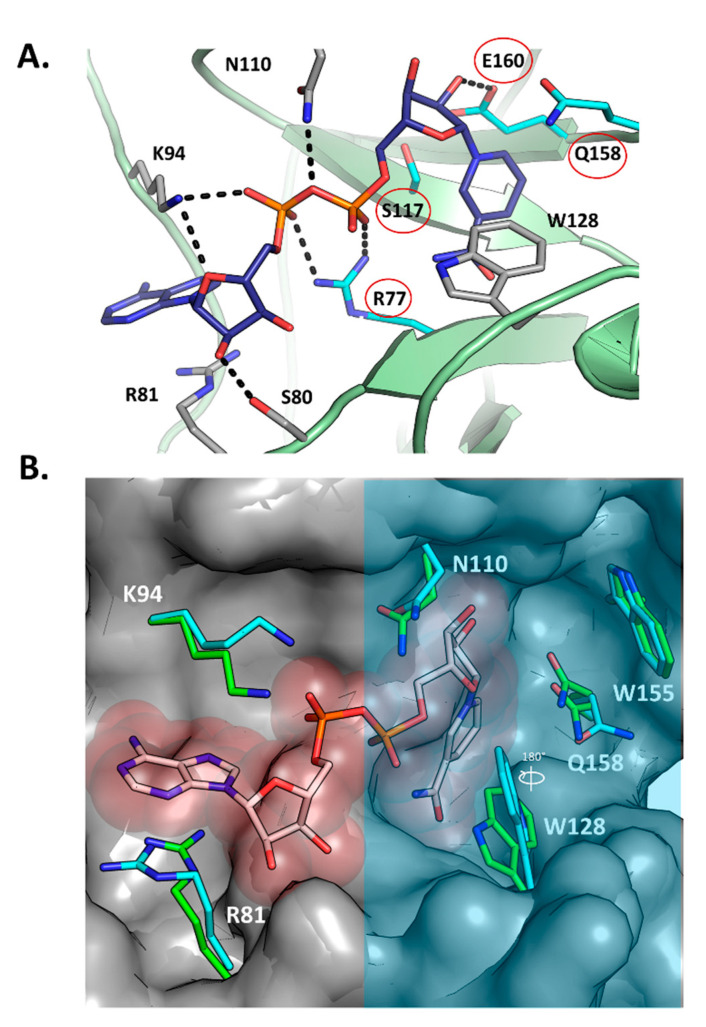
Scabin-NADH interactions. (**A**) The active-site of the Scabin-NADH complex (PDB: 5TLB), showing the H-bond pattern of the active-site residues with NADH. The key residues in the catalytic activity of the enzyme are circled (red) and include R77, S117, Q158, and E160. (**B**) The active-site of the Scabin-NADH complex was overlaid on the apo-Scabin structure (PDB: 5DAZ), showing the conformational changes upon NADH binding. The active-site region that houses the four key members of the Scabin-NAD^+^-DNA bridge is shaded with a translucent light-blue filled rectangle.

Figure 3A shows the structure of Scabin with NADH (PDB: 5TLB) bound in the active site along with the key primary catalytic residues (Arg77, Ser117, Gln158, and Glu160—red circles). Upon NADH binding, the overall substrate pocket architecture of the enzyme is preserved with respect to the apo form (PDB: 5DAZ), according to the small C_α_-RMSD of 0.27 Å (for 24 residues). This fluctuation in the pocket backbone atoms might have its origin in the intrinsic dynamics of the protein since it has the same magnitude as in the entire protein (C_α_-RMSD = 0.27 Å for 165 residues). Notably, upon binding the NAD(H) substrate/inhibitor, the side-chains of the pocket residues are conformationally shifted (RMSD = 0.94 Å, 24 residues), as observed mainly for Arg81, Lys94, Asn110, Trp128, and the catalytic Gln158 (RMSD = 1.76 Å for all-atoms of 5 residues) (Figure 3B). For the catalytic Gln158, two alternate conformations of this side-chain were observed, one that is identical to the apo form and one that is distinct.

A significant conformational change upon NADH binding involves the Trp128 side chain; it appears to have rotated nearly 180°, shifting the nitrogen of the indole ring 4 Å (Figure 3B). The shifts in side-chain location upon NADH binding without changing backbone orientation could explain the relatively high GH catalytic efficiency (specificity constant = 1.4 × 10^6^ M^−1^·min^−1^) of this mART toxin/enzyme. Notably, upon NAD^+^ binding, the ARTT loop of the C3 toxins usually displays large shifts in conformation, signifying an “in” and “out” phase of the loop; however, reported catalytic efficiencies are several orders of magnitudes lower than Scabin for GH activity (C3larvin = 11 M^−1^·min^−1^; C3cer = 2.1 × 10^5^ M^−1^·min^−1^), which suggests that the large structural changes that the C3 toxins employ during catalysis results in relatively inefficient enzymes.

NADH is coordinated in the Scabin active site by a network of hydrogen bonds and steric contacts (Figure 3A), with some similarities and differences in the pattern of interactions described for the bound NAD^+^ substrate in other mART toxins. The nicotinamide amide group is anchored by two reciprocal H-bonds with the backbone of Ser78, which remarkably is a unique substitution for this position into the CT and DT groups (usually Gly, but is a Trp in the Pierisin-like and MTX toxins), and is only found in ScARP (Uniprot: Q9L1E4), a nucleotide-targeting mART from *S. coelicolor* [27,28].

### 2.4. Scabin-NAD^+^-DNA Interface

**Figure 4:**
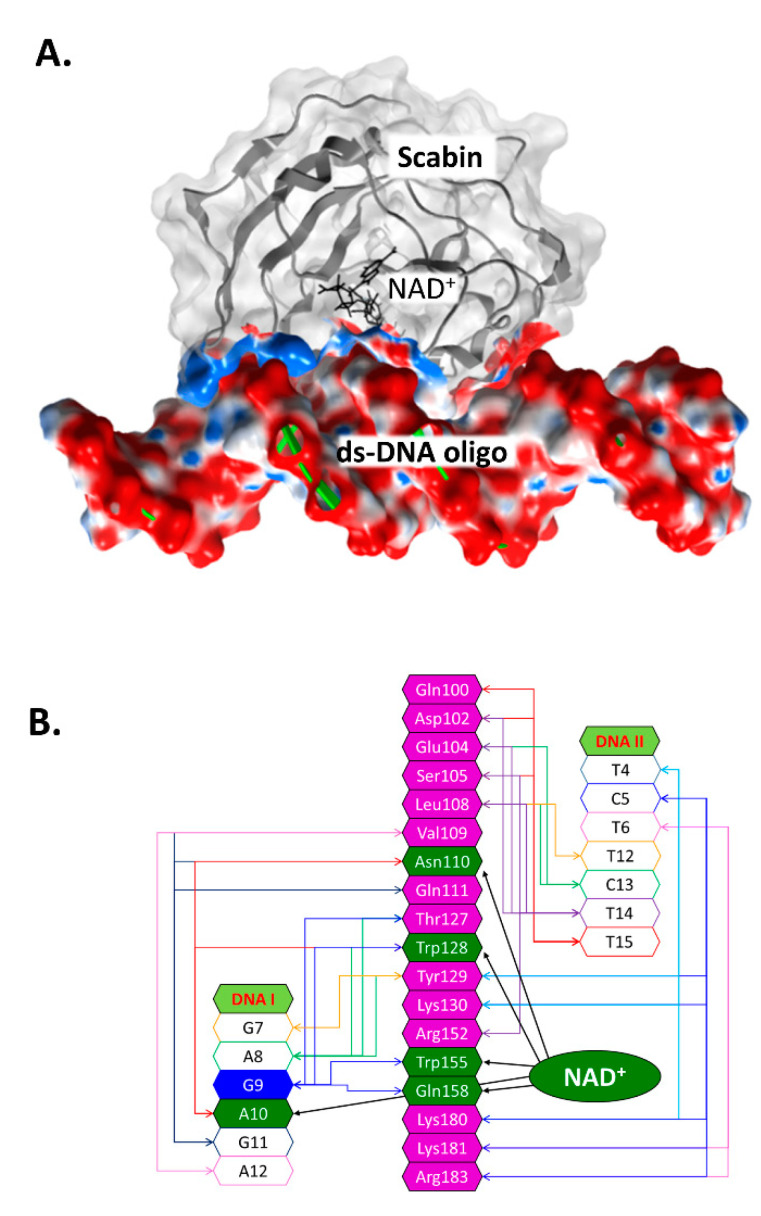
The Scabin-NAD^+^-DNA complex working model. (**A**) The molecular surface of the ternary Scabin-NAD^+^-DNA model is depicted. Scabin is shown in ribbon format with a light gray translucent surface; the contract interface is colored according to its electrostatic potential. NAD^+^ is depicted in black sticks, and the ds-DNA molecule is shown with green ribbons and the surface is also colored by its electrostatic potential. (**B**) Scabin-NAD^+^-DNA interactions. A schematic rendering of the interactions between the Scabin active-site residues (in magenta or dark green) and nucleic acid bases from both DNA strands (unshaded); also shown are the interactions between NAD^+^ and the common residues (in dark green). A central guanine base (G9) is shown in dark blue (9th position of the DNA_I_ strand) and is the target nucleophile in the reaction.

Previously, an in silico Scabin model complexed with NAD^+^ and with a ds-DNA oligomer (21-bp) was prepared by a docking protocol [29]. In the Scabin-NAD^+^-DNA ternary complex (Figure 4A), the solution conformation of Scabin unveils a smaller difference (structural) between the apo-Scabin (PDB: 5DAZ) structure and the enzyme-NADH complex (PDB: 5TLB), with RMSD C_α_ values of 0.88 Å and 0.95 Å, respectively. The NAD^+^ substrate conformation is very similar to NADH in the Scabin-NADH complex (PDB: 5TLB) and to NAD^+^ in the Pierisin-1-NAD^+^ complex (PDB: 5H6J).

According to our model, ds-DNA binds to Scabin with an interface that includes 18 active-site residues and 13 bases on both DNA I and II strands (Figure 4B). Key residues that were identified as important for the interaction of the NAD^+^ substrate include Asn110, Trp128, Trp155, and Gln158 (Figure 2B) [29]. Scabin active-site residues proposed to interact with the ds-DNA substrate include Val109, Asn110, Gln111, Thr127, Trp128, Tyr129, Trp155, and Gln158 (DNA I strand); and Gln100, Asp102, Glu104, Ser105, Leu108, Tyr129, Lys130, Lys180, Lys181, and Arg183 (DNA II strand) [37].

### 2.5. Kinetics of Scabin with Guanine Nucleotides

**Figure 5:**
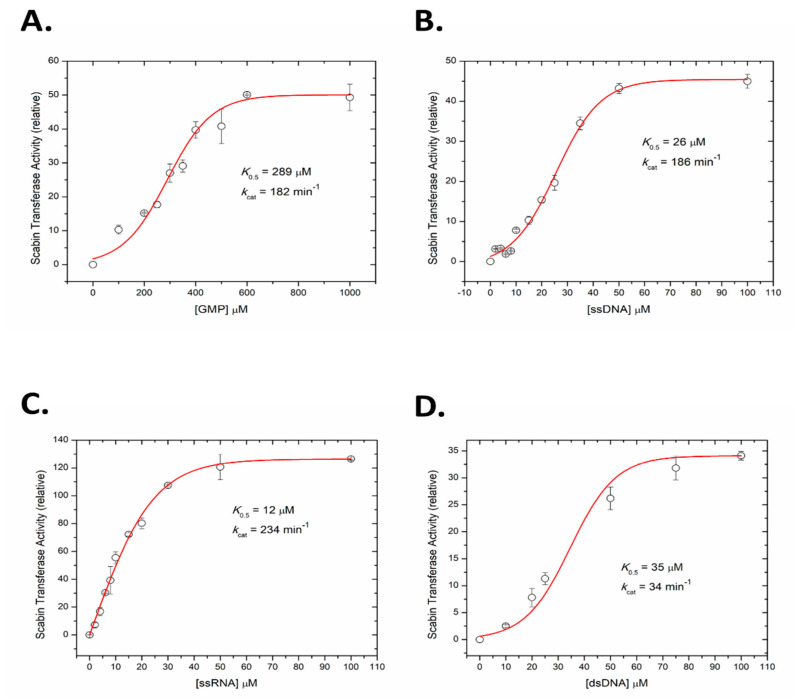
ADP-ribosyltransferase activity of WT Scabin. Scabin WT ADP-ribosyltransferase activity (relative rate) for the transfer of ADP-ribose from NAD^+^ to guanine-containing substrate. (**A**) GMP substrate, (**B**) ss-DNA substrate, (**C**) ss-RNA substrate, and (**D**) ds-DNA substrate. Reaction conditions: Scabin (50 nM), ε-NAD^+^ (250 μM), and various concentration of nucleotide substrates in a 20 mM TRIS pH 7.9 buffer, containing 50 mM NaCl and 1% dimethyl sulfoxide at 25 °C; error bars indicate the SD.

A fluorescence-based assay was developed to measure the kinetics of Scabin ADP-ribosyltransferase activity to guanine nucleotides, including RNA and DNA oligonucleotides. GMP was used as a model nucleotide substrate for Scabin transferase activity and the rate curve against GMP concentration is shown in Figure 5A. The kinetic curve was sigmoidal in nature and the calculated *K*_0.5_ was 289 ± 18 μM with a *k*_cat_ over 180 min^−1^. The kinetic curve for ss-DNA (21-mer with multiple guanine bases, see Materials and Methods for details) is also sigmoidal and is shown in Figure 5B; it reveals that Scabin has a much higher affinity for DNA compared with a single nucleotide substrate such as GMP (*K*_0.5_ = 26 ± 1 μM; *k*_cat_ = 186 min^−1^). The identical nucleotide sequence and length of the ss-RNA was also tested as a Scabin substrate, which showed that Scabin slightly prefers ss-RNA over ss-DNA (*K*_0.5_ = 12 ± 0.5 μM; *k*_cat_ = 234 min^−1^), as shown in Figure 5C. Interestingly, the curve for the ss-RNA substrate was less sigmoidal and nearly hyperbolic in shape (Figure 5C). Further tests showed that Scabin also gave strong kinetic activity with ds-DNA (*K*_0.5_ = 34 ± 2 μM, *k*_cat_ = 34 min^−1^) but the substrate turnover rate was considerably slower than the ss-DNA substrate (Figure 5D). The slower *k*_cat_ for the ds-DNA substrate likely indicates the requirement for separation of the base pairs by the Scabin enzyme and base flipping in ds-DNA for the transfer reaction to the guanine base to occur [39].

### 2.6. Transferase Kinetics of the Scabin Variants

Several site-directed variants of Scabin were prepared based on the Scabin-DNA model shown in Figure 4A,B. This involved residues within the Scabin active-site, including those proposed as DNA-interacting; involving residues Leu108 and Val109 in the α_2_ helix; Asn110 in the L_c_ loop; Trp128, Tyr129, and Lys130 in the PN-loop; Lys154 and Trp155 in the ARTT-loop; Gln158 in the Q-X-E motif in the β_5_ strand; Lys181 and Arg183 found in beta strands 6/7; and Trp199 (control) located outside the catalytic core near the end of the C-terminus (arrows in Figure 1B). Other residues that were studied included two NAD^+^-only interacting residues, such as Ser117 found in the STT(S) NAD^+^ binding motif and Glu160 in the Q-X-E motif. Some active-site residues were proposed to play critical catalytic roles by interacting with both NAD^+^ and DNA, including Asn110, Trp128, Trp155, and Gln158. Conserved residues within the Pierisin family in this study group included Trp68 (structural, folded integrity) and the catalytic hallmark residues Arg77, Asn110, Trp128, Trp155, Gln158, and Glu160.

#### 2.6.1. Kinetics of the Variants of the NAD^+^-Interacting Residues

Table 1 shows the kinetic values of the variants produced from the mutagenesis study of the DNA and NAD^+^-interacting residues. For the NAD^+^-interacting residues (shaded in green in Table 1), the conserved S117 residue was shown to be important for both GH and ADP-ribosyltransferase activity with the Ala substitution variant showing near baseline activities for both catalytic processes. The S-T-S/T motif is known for its role in binding and positioning the NAD^+^ substrate in CT-like mART toxins. Trp68 is outside of the catalytic core of the enzyme but it is conserved within the Pierisin family and likely plays a key structural role to maintain the ADP-ribosyltransferase fold. Ala substitution of Trp68 resulted in a misfolded enzyme protein, probably due to the perturbation of Scabin’s folded integrity, since the variant did not express well and was not very stable in solution. Arg77 is a conserved catalytic residue within all mART family members; replacement of Arg77 with Ala destabilized the Scabin protein and it also could not be tested. Glu160 is the conserved catalytic residue in the Q-X-E motif and its replacement with Ala (in tandem with Gln158), as expected, resulted in both weak GH and transferase activity with 313-fold and 930-fold loss in *k*_cat_ values, respectively.

#### 2.6.2. Kinetics of the Variants of the DNA-Interacting Residues

Ala substitution of the proposed DNA-interacting residues (shaded in yellow in Table 1) showed that Asn110, Trp128, Trp155, and Gln158/Glu160 are the key, primary residues required for catalytic activity involving the transfer of ADP-ribose to water (GH activity) or to the guanine base (ADP-ribosyltransferase), since these variants were severely impaired in both enzymatic activities (Table 1). Lys154 is located close to the major DNA groove and is only 6.8 Å from the guanine G9 base in the DNA substrate of the modelled complex. The K154A variant lost nearly all GH and ADP-ribosyltransferase activity, suggesting that it plays an important role in catalysis, which might be to form critical H-bonds with the DNA bases in ds-DNA in Scabin—Lys154 is only conserved between Scabin and ScARP but not among other Pierisin family members, where it is replaced with a proline (Figure 1B). Both Tyr129 and Arg183 are important for catalytic activity since both the GH and ADP-ribosyltransferase activities were severely compromised in the Ala variants (Table 1). Lys130 participates more in the ADP-ribosyltransferase activity than GH activity since the K130A variant had WT-like GH activity but showed only 42% of WT ADP-ribosyltransferase activity. The α2 residues, Leu108, and Val109 showed importance for both catalytic activities, with the former being more important for GH activity while the latter was more important for ADP-ribosyltransferase activity (Table 1).

### 2.7. Scabin Variant Folded Integrity

**Figure 6:**
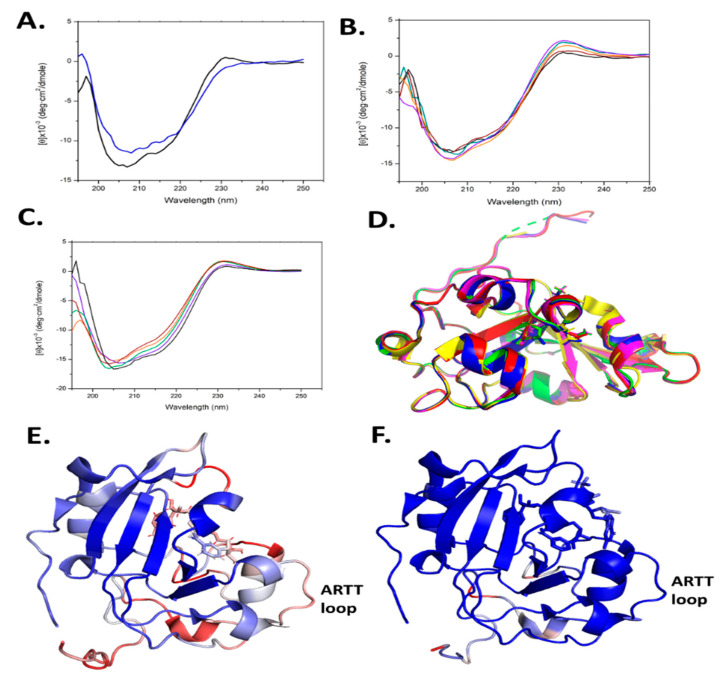
(**A**–**C**) Circular dichroism spectra of WT Scabin and site-directed Tyr129 variants. (**A**) WT (black) and Y129T (blue); (**B**) WT (black), Y129K (orange), Y129H (purple), Y129E (green), and Y129F (maroon). (**C**) WT Scabin (black), K130A (orange), K181A (purple), V109G (green), and L108G (maroon). The concentration of each protein was 0.2 mg/mL in 20 mM TRIS, 50 mM NaF (pH 8.2) at 25 °C. Each spectrum is the average of 9 independent spectra. (**D**) Structural superposition of Scabin WT and variant structures; WT (PDB: 5DAZ, red); V109G (PDB: 6VV4, yellow); N110A (PDB: 6VPA, green); S117A (PDB: 6VUV, blue); and Y129H (PDB: 6VVF, magenta). The full chain RMSD values compared to the apo-Scabin structure ranged from 0.096 to 0.122 Å. (**E**) The Scabin-NADH structure (PDB: 5TLB), colored according to its B-factors: blue (minimum), white (intermediate), and red (maximum), showing the ARTT-loop (V145-E160). The B-factors ranged from 20 to 50. (**F**) The ScARP-NADH-GDP structure (PDB: 5ZJ5) colored according to its B-factors: blue (minimum), white (medium), and red (maximum), showing the ARTT-loop (V149-E164). The B-factors ranged from 20 to 50.

Site-directed mutagenesis was used to prepare several Scabin variants, as mentioned above. However, this approach may often lead to misfolded or partially folded proteins upon recombinant expression and purification. To assess the folded integrity of the Scabin variants, two approaches were taken: (i) determination of the X-ray crystal structure where possible (apo-WT, PDB: 5DAZ; V109G, PDB: 6VV4; N110A, PDB: 6VPA; S117A, PDB: 6VUV; W128Y, PDB: 6APY; Y129H, PDB: 6UVF; and W155A, PDB: 5UVQ); and (ii) circular dichroism (CD) spectroscopy was conducted to assess the solution’s secondary structure. The CD results for some of the variants were previously reported [20,29] and the CD spectra for the remaining variants are shown in Figure 6A–C. It is noteworthy that the R77A and W69A variants could not be studied for catalytic activity since they were not properly folded (data not shown). In addition, the Y129T variant showed some perturbation in its CD spectrum (Figure 6A). The rest of the Scabin variants showed either a WT-like crystal structure (Figure 6D) [20,29] or similar CD spectra to the WT in solution (Figure 6B,C) [20,29].

### 2.8. Scabin Crystal Structures

The structure of recombinant Scabin was previously reported at a 1.50 Å resolution in the apo (substrate-free) form and with two good NAD^+^ competitive inhibitors bound in the active site [20]. Later, our group also solved the structure of Scabin with NADH, as a substrate analog of NAD^+^, providing a reasonable model for the Scabin-NAD^+^ Michaelis complex [37]. In addition, we solved the structures of the Scabin variants for two conserved Trp residues, W128Y and W155A, which are kinetically compromised variants of the enzyme, suggesting the key catalytic role that these conserved indoles play in the enzyme mechanism [37].

Several small molecules were previously shown to inhibit Scabin GH activity. PJ34, P6-C, P6-D, P6-E, and P6-F were previously shown as inhibitors of mART toxins [3,40], and the results showed that Scabin has an NAD-binding pocket similar to the CT-like mART toxin family [29]. In the present work, we solved the crystal structures of another four catalytic variants, V109G (PDB: 6VV4), N110A (PDB: 6VPA), S117A (PDB: 6VUV), and Y129H (PDB: 6VVF), and the crystallographic data and refinement statistics are shown in Table S1. All the structures were of high quality, ranging from 1.5 to 1.75 Å. The structures of all four variants with Scabin were superposed in Figure 6D and all four structures were properly folded, as revealed by the RMSD values for each variant’s main chain compared with the WT (PDB: 5DAZ) structure (RMSD values ranged from 0.096 to 0.122).

### 2.9. Key Residues for the DNA-Substrate Interaction and ADP-Ribosylation of the DNA Substrate

#### 2.9.1. Scabin N110A Variant

It is clear from the kinetic data in Table 1, with ss-DNA substrate, that N110 is a key, conserved active-site residue that interacts with DNA and participates during the catalytic cycle. Three residues (Trp128, Trp155, and Gln158) bridge the NAD^+^ and DNA substrates and these were studied and compared to the Scabin WT enzyme [29]. Herein, the fourth-member of this proposed Scabin-NAD^+^-DNA bridge, Asn110, was investigated with the N110A variant. Regarding the NAD^+^ substrate, the affinity was previously estimated by measuring the *K*_M_ of the GH activity using the εNAD^+^ analog [20,29]. In Scabin, the Asn to Ala substitution does not perturb enzyme interaction with the NAD^+^ substrate, since the Michaelis-Menten constant of the N110A variant, *K*_M_(εNAD^+^)_N110A_ = 66 ± 12 μM, is nearly identical to the WT value—*K*_M_(εNAD^+^)_WT_ = 68 ± 3 μM. Regarding the ds-DNA substrate, the affinity was determined by measuring the binding constant of a ds-DNA tagged with cyanine-3. In Scabin, the Asn to Ala substitution modestly reduces the interaction with ds-DNA, since the dissociation constant of the N110A variant, *K*_D_(21bp-DNA)_N110A_ = 82 ± 5 μM, is higher than the K_D_ of the WT toxin of *K*_D_(21bp-DNA) = 51 ± 4 μM. The small effect of substitution with Ala on DNA-binding affinity (1.5×) may reflect the small contribution of the Asn110 H-bond to the binding energy. Notably, replacement of Asn110 with Ala severely compromised the enzyme’s ability to transfer ADP-ribose to ss-DNA (Table 1), making Asn110 an important DNA-transferase residue in Scabin and the fourth member of the Scabin-NAD^+^-DNA bridge. Given its conservation among the Pierisin family (Figure 1B), Asn110 is the new catalytic cornerstone for DNA substrate modification.

#### 2.9.2. Scabin Trp155A Variant

The modest impairment (3×) in ability of the W155A variant to bind DNA [29] also agrees with the Trp155 associations seen in the Scabin⋅DNA model since it reveals surface van der Waals contacts with the DNA_I_ backbone. However, Trp155 is essential for the transferase activity towards both deoxyguanosine and guanine-containing ds-DNA substrates (Table 1 and [29]). Considering the role of Trp155 activity and its participation as a key ARTT-loop residue(s) in mART toxins, in general [33,34,41,42], we earlier proposed that Trp155 stabilizes the reactive guanine base for the transfer reaction; i.e., Trp155 interacts with the reactive guanine base in the DNA substrate [29]. Recently, the structure of ScARP with NADH and GDP revealed that the guanine base in GDP stacks between the N-ribose of NADH and Trp159 (Trp155 in Scabin) [28]. This conserved Trp residue forms H-bonds with the N^2^ and N^3^ of guanine in GDP and the center of this indole ring is 4.4 Å from the center of the guanine base in GDP and the indole side chain is only 7.4 Å apart from the C-atom of the glycosidic ribose-nicotinamide bond [28]. This positions the nucleophilic NH_2_ of guanine to about 4.0 Å from the electrophilic C in the N-ribose of NAD(H), which makes it ripe for the ADP-ribosyltransferase reaction with the GDP substrate. However, caution is required since the binding of the DNA oligonucleotide (either ss- or ds-DNA) may alter the geometry of the catalytic residues in the reaction center and no structure of any Pierisin family member with the true Michaelis NADH-DNA complex has yet been determined.

#### 2.9.3. Scabin K154A Variant

The K154A variant was highly compromised in both GH and ADP-ribosyltransferase activity (Table 1). Interestingly, Lys154 is not conserved in the Pierisin family but is shared with ScARP (Figure 1B); it is close to the major DNA groove in the Scabin-NAD-DNA model and 6.8 Å from the G9 base. Upon considering the flexibility of the Lys154 side chain and its H-bond capability along with its charged polar head group, its role in catalysis may be to disrupt the H-bonds in paired DNA bases, where a low dielectric environment—reinforced by Leu108 (located in α_2_)—facilitates the electrostatic character of the disrupting interaction(s). It may also provide a stable electrostatic environment for guanine small nucleotide substrates.

#### 2.9.4. Scabin W128Y Variant

Trp128 is absolutely conserved throughout the Pierisin family and is found in the catalytic PN-loop (Figure 1B). Clearly, it is a key catalytic residue involved in both the GH and ADP-ribosyltransferase activities, as evidenced by the near total loss of both enzymatic activities upon replacement with tyrosine (Table 1). Upon binding NADH, Trp128 adopts a different conformation to that seen in apo-Scabin (Figure 3B). The PN-loop region in apo- and NADH-bound Scabin is quite disordered, exhibiting poor electron density throughout, indicating that this catalytic loop is poised for interaction with the DNA substrate (Figure 6E). The corresponding PN-loop in ScARP is more ordered/less mobile and the ScARP protein is much less flexible/mobile overall (Figure 6F) [28]. The Pierisin-1-NAD^+^ structure shows even greater mobility (higher B-factors than Scabin) and it also has a highly mobile and much larger PN-loop in the NAD^+^ bound structure [32]. In the ScARP-NADH-GDP structure, the side chain of this conserved Trp (Trp132 in ScARP) moves from the apo- to the GDP-bound state to accept the guanosine ribose in the GDP substrate [28]. Thus, the role of the Trp in the PN-loop is to receive and stabilize the guanosine ribose during the ADP-ribosylation event in GDP and likely in DNA as well.

#### 2.9.5. Scabin Y129(X) Variants

Previously, Tyr129 was shown to be important for DNA binding, but was not involved in the reaction mechanism [29]. According to our Scabin-NAD^+^-DNA model, Tyr129 is important for binding DNA [29], based on its location and orientation in the complex. Our model suggests that Tyr129 reaches into the DNA minor groove and contacts both the DNA I and II strands. However, binding of DNA substrate is likely controlled by a rather large contact area and contributions from several backbone and side-chains within the Scabin active site. The kinetic data for the Y129A variant showed that there was only a modest effect of tyrosine replacement on the ADP-ribosyltransferase activity of Scabin (Table 1). A similar reduction was observed for Scabin GH activity. Several replacements at Tyr129 were made to ascertain the chemical nature of the Scabin-DNA interaction. Replacement of Tyr129 with a negative charge (Y129E) showed the largest effect on ADP-ribosyltransferase activity (21% of WT activity) (Table 1). This may be explained by the negatively charged Glu129 exerting electrostatic repulsion with the phosphate groups of the DNA substrate.

#### 2.9.6. Scabin Q158A/E160A Variant

Clearly, the conserved residues, Gln158 and Glu160, play key roles in the enzyme mechanism of the Pierisin family members, including Scabin. Replacement of these residues with Ala nearly obliterated both the GH and ADP-ribosyltransferase enzyme activities (Table 1). As reported by Yoshida and Tsuge (2018), the Gln162 (Gln158 in Scabin) forms H-bonds with the N^2^ and N^3^ atoms of guanine in the GDP substrate [28]. The conserved Glu residue in all mART toxins (Glu-160 in Scabin) H-bonds the N-ribose of NAD^+^ and serves to facilitate the C-N bond cleavage to form the reactive oxocarbenium intermediate [43,44]. In Pierisin family enzymes, the role of this hallmark Glu is to orient the N^2^ of guanine and to steer the lone pair orbital density of N^2^ towards NC1 in the NAD^+^ substrate [28].

### 2.10. ADP-Ribosylation of DNA

**Figure 7:**
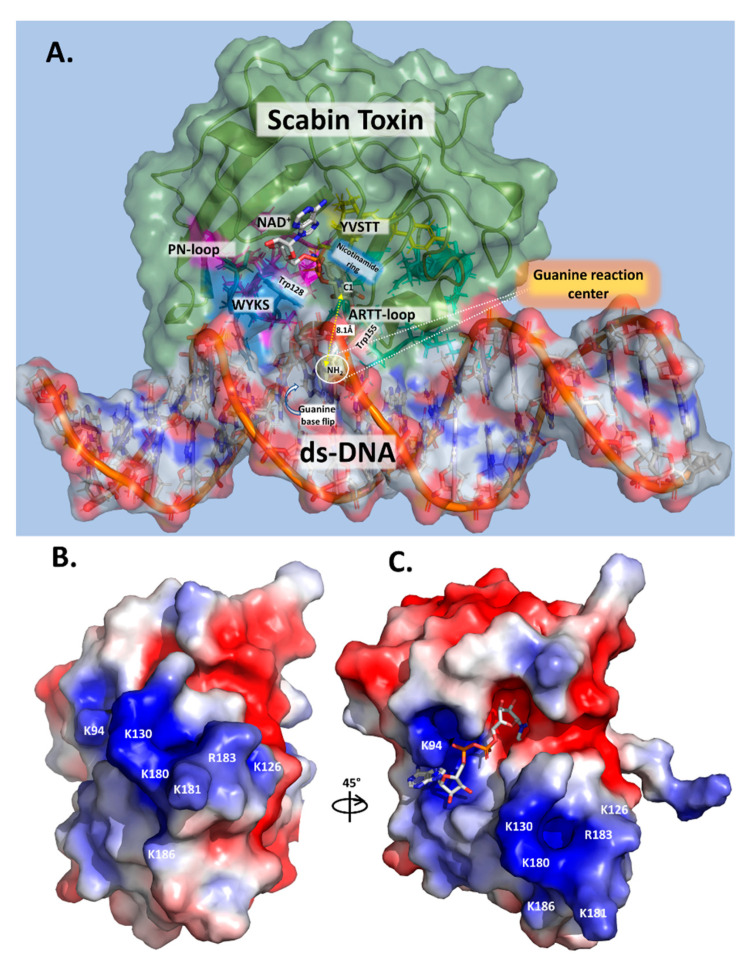
Scabin models. (**A**) Scabin-NAD^+^-ds-DNA model. The Scabin-NADH structure (PDB: 5TLB) was colored in pale green (50% translucent) and docked with ds-DNA (21 base pair oligonucleotide, colored according to electrostatic potential) in a surface representation. The reactive guanine-9 base was flipped out from the DNA duplex into the active site pocket of the Scabin-NADH and the key catalytic elements are shown. (**B**) Surface potential model of Scabin, showing the face of the proposed DNA-binding site. Molecular surfaces are colored by the relative electrostatic potential (red—negative or acidic; blue—positive, or basic). Basic residues (shown in blue) are labelled and shown on the surface of the proposed DNA-binding site. (**C**) Surface potential of the Scabin-NADH rotated approx. 45° to show the active-site pocket. Basic residues within the proposed DNA-binding site, as shown in (**B**), are also depicted in this view.

Based on these results, a mode of DNA substrate binding for Scabin is proposed, which represents a recognition motif for DNA-targeting bacterial mART toxins (Figure 7A). The key structural motifs are shown in the current Scabin-NAD-DNA model and the mechanism involving the ds-DNA substrate involves a base-flip of the target guanine, as observed in the DNA repair enzymes such as O(6)-alkylguanine-DNA alkyltransferase (AGT) [45,46] (Figure 7A). Trp128 and Tyr129 interact with adjacent nucleotides to the guanine nucleophile, allowing Trp155 to dock to the target guanine base. The target guanine then moves into position and is recognized by Gln158, like the proposed recognition of Asn by Gln212 of C3 exoenzyme [12]. Gln158 allows for the guanine nucleophile to move near the glycosidic bond of NAD^+^, ready for ADP-ribosylation. Glu160 stabilizes the oxocarbenium ion intermediate, whereby the C1 of N-ribose undergoes nucleophilic attack by the N2 exocyclic amine of the guanine base [12]. The mechanism ends with the formation of an ADP-ribosylated guanine base within the DNA. Additionally, Scabin has been shown to bind single-stranded breaks in ds-DNA [20], similarly to poly-ADP-ribosylpolymerase-1 (PARP-1) [47]. However, Scabin possesses no sequence or structural similarities to PARP-1 beyond the classic catalytic motifs present in ADP-ribosyltransferases. Therefore, Scabin is clearly a unique enzyme among members of both poly-ADP-ribosyltransferases and the bacterial mono-ADP-ribosyltransferase families. It has strong substrate preferences for mononucleotides and both RNA and DNA (Figure 5), which also makes it unique among Pierisin family members.

### 2.11. The Scabin-NAD^+^-DNA Complex in the Context of Substrate Binding

As previously reported, the ARTT- and PN-loops participate with both the NAD^+^ and macromolecule substrates during catalysis in mART toxins [34,41,43]. The ARTT-loop, aside from the semi-conserved Trp155 (W_ARTT_) and Gln158 (Q_ARTT_), provide a unique sequence with non-conserved substitutions. The model and experimental data concur that Scabin Trp155 is important in binding the DNA substrate. However, the DNA affinity in Pierisin-1 was not significantly affected in the W160A variant compared to the WT protein [32]. Trp155 is close (<4 Å) to NADH (PDB: 5TLB) and NAD^+^ (this work) in Scabin, while its counterpart, Trp160, is farther (>6Å) from the bound NAD^+^ in Pierisin-1 (PDB: 5H6J). In Scabin, Arg152 and Lys154 participate in the interactions with ds-DNA substrate. These data suggest an important role of the ARTT-loop in Scabin and other members of the Pierisin-like group, in both NAD^+^ and DNA substrate binding and stabilization.

## 3. Conclusions

In the Pierisin-like mART toxin group, the sequence motif R_β1_-R_La_-N_Lc_-STT_β2_-W_PN_-W_ARTT_-(QxE)_ARTT_ appears as a signature motif involved in the mART catalytic function of these proteins/toxins. Importantly, (i) the reported DNA-contacting residues/motifs in Pierisin-1 are not present in Scabin; and (ii) many of the proposed DNA-contacting residues/motifs in Scabin are not present in other members of the Pierisin-like group. Thus, it may be concluded that Scabin possesses a unique DNA-binding motif within the Pierisin family. Scabin has a positively charged surface that serves as a DNA/RNA binding surface, which can be seen in Figure 7B,C, and it is largely shared with ScARP but no other Pierisins (Figure 1B). The Scabin central core structure is like that of ScARP, Pierisin-1, and other ADP-ribosyltransferases [32]. However, Scabin and ScARP do not possess either a ricin B-like domain or an autoinhibitory linker as found in Pierisin-1, implying that enzyme regulation is different between Scabin/ScARP and Pierisin-1. The arrangement of the α-helices and loop regions around the core scaffold is also different in Scabin/ScARP and their PN-loops are much smaller than in Pierisin-1 (Figure 1B) [28,32]. The key positively charged residues for DNA binding are also not conserved between Pierisin-1 and Scabin/ScARP (Figure 1B, filled black circles; Figure 7B,C) [32]. These observations indicate that the binding mode and preference for various guanine-containing substrates differ between Scabin/ScARP and Pierisin-1. Clearly, the DNA-binding and targeting mechanisms, including biological roles, are not universal among the Pierisin family members. Structural elucidation of the full Michaelis complex of various Pierisin family members with both NAD^+^ and ds- and ss-DNA substrates is the next step towards a better understanding of the reaction mechanism of the DNA-targeting ADP-ribosyltransferases.

## 4. Materials and Methods 

Materials: Unless otherwise noted, chemicals were purchased from Sigma-Aldrich (St. Louis, MO, USA), and OriginPro 8.0 (Originlab Corp., Northampton, MA, USA) was used for data fitting and plotting.

### 4.1. Scabin Expression and Purification

The Scabin gene was cloned into a pET-TEV vector containing an N-terminal His_6_ tag with a tobacco etch virus protease cut site. Site-directed mutants were prepared using the Quikchange™ mutagenesis method [48]. Chemically competent *Escherichia coli* BL21 λDE3 cells were transformed with plasmid and grown overnight at 37 °C on LB media containing 30 μg/mL kanamycin. Half the colonies on each plate were scraped into 50 mL of LB containing kanamycin and allowed to grow to an OD of 0.6 at 37 °C with shaking; 25 mL of culture was inoculated into 800 mL of 2xYT media containing kanamycin. Cells were grown to an OD of 1.2 at 37 °C with shaking and subsequently induced with 1 mM isopropyl β-D-1-thiogalactopyranoside for 4 h. Wild-type and variants were all expressed using the above conditions, except for Q_158_A/E_160_A and N_110_A, which yielded better expression when induced for 16 h at 16 °C. Cells were pelleted at 4000× *g* and resuspended in lysis buffer containing 25 mM Tris-HCl, pH 8.2, 200 mM NaCl, 50 μg/mL CHAPS, 120 μM PMSF, 1 mM EDTA, and 100 μg/mL DNase. Resuspended cells were lysed using an Emulsifex-C3 high-pressure homogenizer (Avestin Inc., Ottawa, ON, Canada) and subsequently centrifuged at 14,000× *g* for 50 min at 4 °C. Protein was purified from whole cell lysate using immobilized metal-affinity chromatography. The supernatant was passed over a HiTrap IMAC HP 5 mL column (GE Healthcare, Mississauga, ON, Canada) equilibrated with 5 mM imidazole in binding buffer (50 mM TAPS, pH 8.5, 500 mM NaCl). The column was washed with 25 mM imidazole in binding buffer and the protein was eluted using a gradient from 25 to 250 mM imidazole. Fractions containing the protein of interest were resolved on an SDS-PAGE gel to confirm its identity; the purified protein was pooled and dialyzed into 50 mM Tris-HCl, pH 8.2, and 50 mM NaCl, deemed dialysis buffer 1.

Further purification was performed via anion-exchange chromatography. Briefly, a HiTrap Q-Sepharose HP column was equilibrated with dialysis buffer 1 and the protein sample was passed over the column; bound protein was subsequently eluted with a linear gradient from 50 to 500 mM NaCl in dialysis buffer 1. Fractions that contained purified protein, as confirmed by SDS-PAGE, were pooled, and concentrated initially on a bed of PEG 20,000 at 4 °C to reduce the mechanical stress on the protein and for optimal recovery. Once the sample was concentrated to approximately 5 mL, the remainder was brought to 1.5 mg/mL using Millipore 0.5-mL 10-kDa spin columns at 3000× *g* for 10-min intervals in a microfuge; the protein yield was approximately 1 mg per liter of culture.

### 4.2. Circular Dichroism Spectra

Circular dichroism (CD) spectra were acquired for all Scabin variants using a JASCO J-815 CD spectropolarimeter (Easton, MD, USA) (250–195 nm scan, average of 9 spectra). The protein was at 0.16 mg/mL in a buffer containing 20 mM Tris-HCl, pH 8.2, and 50 mM NaF in a 1-mm path length quartz CD cuvette.

### 4.3. Protein Crystallography

Crystal conditions for Scabin were as previously reported [20]. X-ray diffraction data were obtained at the Canadian Light Source in the Canadian Macromolecular Crystallography Facility (beamline, 08ID-1).

### 4.4. Scabin Structures

Collected data were processed in XDS [35]. Molecular replacement was performed using Phenix [49] with the Scabin-apo (PDB: 5DAZ) structure as the model. Iterative cycles of model building was performed in COOT [50] and subsequent refinement in Phenix. The Scabin V109G, N110A, S117A, and Y129H structures were deposited in the Protein Data Bank database, with the codes 6VV4, 6VPA, 6VUV, and 6VVF, respectively. Table S1 shows the crystallographic details for these structures.

### 4.5. GH Activity

Glycohydrolase assays were conducted with a Cary Eclipse fluorescence spectrophotometer (excitation wavelength, 305 nm; emission wavelength, 405 nm; and band passes of 5 nm) [51]. ε-NAD^+^ substrate (0–450 μM) were mixed with 490 nM Scabin in NAD^+^ GH buffer consisting of 20 mM Tris-HCl, pH 7.9, and 50 mM NaCl. Triplicate reactions were measured for 10-min intervals and the reaction initial slope was recorded. An εAMP standard curve was obtained to convert the fluorescent values to εADP-ribose formed per min. A Michaelis-Menten curve was plotted and fitted with OriginPro-8 software using a hyperbolic model (OriginLab Corp., Northampton, MA, USA).

### 4.6. ADP Ribosyltransferase Reaction

A novel ADP-ribosyltransferase assay with GMP, ss-RNA, and ss- and ds-DNA was performed, as described under “GH activity”, with some important modifications. This fluorescence-based method allows for the acquisition of time-based kinetic data using a fluorescence spectrophotometer and can be generally applied to other DNA-modifying ADP-ribosyltransferases. A Varian Eclipse fluorescence spectrophotometer was set to λ_ex_ at 320 nm with a 1.5 nm bandpass and λ_em_ at 405 nm with a 20 nm bandpass. This enabled excitation of the etheno fluorescence in ε-NAD^+^ without inner filter interference from the UV absorbance of the nucleic acids in the GMP, RNA, or DNA, since nucleic acids absorb weakly (near zero) at 320 nm. The ε-NAD^+^ was held at a concentration of 250 μM and was mixed with 50 nM Scabin in a GH buffer containing 1% dimethyl sulfoxide and various concentrations of GMP (0–1000 μM), ss-RNA (0–100 μM), and ss- or ds-DNA (0–100 μM). The data were fit to the sigmoidal kinetic model in OriginPro-8 (OriginLab Corp., Northampton, MA, USA).

### 4.7. Modeling the Scabin-NAD^+^-DNA Complex

The Scabin-NADH X-ray structure (PDB: 5TLB) was stripped of crystallographic water molecules and NADH was crafted to NAD^+^ in situ. The MOE Amber12: EHT force-field was used to prepare the NAD^+^ molecule. The whole molecular system was treated with the Protonate3D protocol while protecting the oxidized state of the ligand, but protecting the oxidized state of the ligand. In the resultant system, the NAD^+^ molecule (10 kcal/mol, 0.25 Å buffer) and the heavy atoms (100 kcal/mol), except for the side-chains atoms at ≤4.5 Å from the nicotinamide moiety, were re-organized with energy minimization until an RMSG ≤ 0.001 kcal/mol/Å^2^ was obtained. The 21-base pair double-stranded DNA oligo, 5′-GGAAGAGAGAGAGAAAGAGAG-3′ (forward strand), was assembled in a B-helix conformation. A coarse-grained MOE protein-protein docking of the ds-DNA molecule as a substrate onto the Scabin-NADH (receptor) was performed. This approach uses rigid side-chains and an implicit solvent model. The ternary decoys (30 highest ranked) were iteratively optimized by implementing and then alternating the “rigid-body” energy minimization (RMSG ≤ 0.1 kcal/mol/Å^2^) of the DNA onto a fixed Scabin-NAD^+^ structure with “conformational” energy minimization (RMSG ≤ 0.01 kcal/mol/Å^2^). This was followed by relaxation of the free interfacial atoms (side-chains and loop atoms in contact with DNA) and was restrained with 10 kcal/mol neighbors (≤4.5 Å from the interfacial atoms) until the total energy difference between the two consecutive iterations was less than 0.1 kcal/mol. The lowest energy decoy was saved as the Scabin-NAD^+^-DNA ternary complex model.

### 4.8. Availability of Data and Materials

The Scabin variant X-ray structures were deposited at the Protein Data Bank (http://www.rcsb.org/pdb): V109G (PDB: 6VV4), N110A (PDB: 6VPA), S117A (PDB: 6VUV), and Y129H (PDB: 6VVF).

## Figures and Tables

**Table 1:** Kinetic parameters of the Scabin WT and variants for GH and ADP-ribosyltransferase activity with the ss-DNA substrate.

Scabin Protein	^a^ GH Activity		^b^ ADP-Ribosyltransferase Activity
	*K*_M_ (μM)	*k*_cat_ (min^−1^)	*k*_cat_ (min^−1^)
WT	68 ± 3	94 ± 5	186 ± 8
L108G	49 ± 8	11 ± 0.7	71 ± 3
V109G	111 ± 23	55 ± 6	16 ± 1
N110A	66 ± 12	1 ± 0.05	31 ± 2
S117A	88 ± 14	1.3 ± 0.16	15 ± 1
W68A	^c^ ND	27 ± 3	93 ± 3
W128Y	17 ± 3	1 ± 0.01	7.5 ± 1
W155A	55 ± 10	10 ± 0.4	5.5 ± 0.3
W199A	40 ± 7	12 ± 0.12	177 ± 2
Y129A	86 ± 11	60 ± 3	119 ± 8
Y129E	51 ± 9	117 ± 4	40 ± 1
Y129F	66 ± 9	57 ± 2	^c^ND
Y129H	25 ± 5	55 ± 3	90 ± 5
Y129K	67 ± 6	61 ± 2	132 ± 3
Y129T	^c^ND	23 ± 2	121 ± 2
K130A	160 ± 19	141 ± 5	79 ± 4
K154E	97 ± 7	18 ± 2	20 ± 1
K181A	33 ± 3	32 ± 3	74 ± 3
R183A	^c^ND	40 ± 3	58 ± 2
Q158A/E160A	53 ± 9	0.3 ± 0.02	0.2 ± 0.01

^a^ GH activity is the hydrolysis activity of the NAD^+^ substrate in the absence of a nucleotide substrate. ^b^ ADP-ribosyltransferase activity is the transfer of ADP-ribose from NAD^+^ to the ss-DNA substrate (21 bp, see Materials and Methods for sequence information); the experiments for enzyme activity represent the mean ± SD of at least three different measurements. ^c^ ND, not determined. The variants are colored according to their involvement in NAD^+^ substrate interactions (green), DNA substrate binding (orange), or DNA transferase activity (yellow). Residues without color are not involved in the catalytic mechanism of the enzyme and/or indicate a role in structural integrity.

## Data Availability

Data are available upon request, please contact the contributing authors.

## Supplementary Materials

The following are available online at https://www.mdpi.com/2072-6651/13/1/55/s1, Table S1: Crystallographic data and refinement statistics for Scabin catalytic variant structures.
