# Supplementary material for: Mapping the DNA-Binding Motif of Scabin Toxin, a Guanine Modifying Enzyme from Streptomyces scabies
Source: Toxins (Basel). 2021 Jan 13;13(1):55. doi: 10.3390/toxins13010055 (PMC7828395; doi:10.3390/toxins13010055)
Supplement: Supplementary file 1 [file toxins-13-00055-s001.pdf]

Supplementary Materials: Mapping the DNA-Binding Motif of Scabin Toxin, a Guanine Modifying Enzyme from *Streptomyces scabies*

Maritza Vatta, Bronwyn Lyons, Kayla A. Heney, Taylor Lidster and A. Rod Merrill

Table S1. Crystallographic data and refinement statistics for Scabin catalytic variant structures.

Scabin Variant	N110A	V109G	Y129H	S117A
PDB ID	6VPA	6VV4	6VVF	6VUV
X-ray source	CLSI-08-ID-1	CLSI-08-ID-1	CLSI-08-ID-1	CLSI-08-ID-1
Wavelength (Å)	0.97949	0.97949	0.97949	0.97949
Unit cell parameters (Å)	a = 88.4, b = 60.7, c = 37.9<br>α = 90.0, β = 99.4, γ = 90.0	a = 88.6, b = 60.2, c = 37.9<br>α = 90.0, β = 99.4, γ = 90.0	a = 87.9, b = 61.1, c = 38.0<br>α = 90.0, β = 99.9, γ = 90.0	a = 88.5, b = 60.9, c = 38.0<br>α = 90.0, β = 99.6, γ = 90.0
Space group	C2	C2	C2	C2
Resolution range (Å)[a]	31.35–1.50	26.37–1.75	37.4–1.7	43.61–1.55
Data completeness (%)	99.2 (93.2)	99.7 (99.1)	98.1 (88.1)	99.7 (99.6)
R_{merge}	0.0424 (0.3464)	0.04077 (0.7059)	0.03897 (1.178)	0.04171 (0.9209)
Redundancy	4.5 (3.9)	3.8 (3.7)	3.7 (2.9)	4.2 (4.1)
Average I/σ(I)	20.2 (3.8)	18.5 (2.1)	19.1 (1.0)	17.7 (1.6)
Molecular replacement program	Phaser	Phaser	Phaser	Phaser
R_{work} (%)[b]	14.46	17.88	17.95	17.86
R_{free} (%)[c]	17.21	20.53	21.00	21.01
No. of atoms in protein	1309	1319	1272	1300
No. of waters	123	117	110	156
Root mean square deviation from ideal bond length (Å)	0.010	0.004	0.020	0.005
Root mean square deviation from ideal bond angle (°)	1.06	0.71	1.48	0.76
B-Factors (Å^2) for protein	25.20	31.19	32.92	31.22
B-Factors (Å^2) for water	37.06	36.60	38.04	45.40
Ramachandran plot favored (%)	96.89	96.32	96.23	96.32
Ramachandran plot outliers (%)	0.62	0.61	0.63	0.61

[a] Values in parenthesis are for the highest resolution shell; [b] $\sum||Fobs|-|Fcalc|| \sum|Fobs|$, where $|Fobs|$ and $|Fcalc|$ are the observed and calculated structure factor amplitudes, respectively; [c] The R_{free} value was calculated with a random 5% subset of all reflections excluded from refinement.
